# Recent Progress in Photocatalytic Degradation of Water Pollution by Bismuth Tungstate

**DOI:** 10.3390/molecules28248011

**Published:** 2023-12-08

**Authors:** Yingjie Zhang, Huijuan Yu, Ruiqi Zhai, Jing Zhang, Cuiping Gao, Kezhen Qi, Li Yang, Qiang Ma

**Affiliations:** 1 College of Agriculture and Biological Science, Dali University, Dali 671000, China; yjzhang_dlu@163.com (Y.Z.); hjyu_yhj@163.com (H.Y.); zhairuiqi@163.com (R.Z.); zj2452488891@163.com (J.Z.); gaocp_dlu@163.com (C.G.); 2 Key Laboratory of Ecological Microbial Remediation Technology of Yunnan Higher Education Institutes, Dali University, Dali 671000, China; 3 College of Pharmacy, Dali University, Dali 671000, China; 4 College of International Education, Dali University, Dali 671000, China; yangliisme@163.com; 5 School of Architecture and Civil Engineering, Chengdu University, Chengdu 610106, China

**Keywords:** Bi_2_WO_6_, photocatalyst, waste water, morphology control, composite fabrication

## Abstract

Photocatalysis has emerged as a highly promising, green, and efficient technology for degrading pollutants in wastewater. Among the various photocatalysts, Bismuth tungstate (Bi_2_WO_6_) has gained significant attention in the research community due to its potential in environmental remediation and photocatalytic energy conversion. However, the limited light absorption ability and rapid recombination of photogenerated carriers hinder the further improvement of Bi_2_WO_6_’s photocatalytic performance. This review aims to present recent advancements in the development of Bi_2_WO_6_-based photocatalysts. It delves into the photocatalytic mechanism of Bi_2_WO_6_ and summarizes the achieved photocatalytic characteristics by controlling its morphology, employing metal and non-metal doping, constructing semiconductor heterojunctions, and implementing defective engineering. Additionally, this review explores the practical applications of these modified Bi_2_WO_6_ photocatalysts in wastewater purification. Furthermore, this review addresses existing challenges and suggests prospects for the development of efficient Bi_2_WO_6_ photocatalysts. It is hoped that this comprehensive review will serve as a valuable reference and guide for researchers seeking to advance the field of Bi_2_WO_6_ photocatalysis.

## 1. Introduction

With the rapid growth of the global economy and population, water pollution has become an increasingly serious challenge. Solving water pollution problems through the effective utilization of natural resources for economic sustainable development has become the focus of scientific researchers [1,2,3]. The main source of water pollution is the discharge of domestic, agricultural, and industrial sewage [4]. This sewage contains a large amount of heavy metal and organic pollutants. Heavy metals like Hg and Cr are toxic and not easily broken down by the environment, which negatively impact the living environment of aquatic animals and plants [5]. Furthermore, heavy metals can accumulate in the food chain, affecting the growth and development of organisms in the water and leading to stagnation or even death [6]. Common organic pollutants found in sewage include phenolic compounds, aniline compounds, and organophosphorus pesticides. Organic pollutants pose serious hazards to the environment and organisms, lead to contamination of water bodies and soil, exerting toxic effects on plants and animals in the ecosystem, thus disrupting the ecological balance. They may also contaminate agricultural irrigation water, adversely affecting crop growth. Furthermore, certain organic pollutants have the potential to accumulate within organisms and gradually concentrate through the food chain, posing hazards to higher level animals in the food web [7].

The degradation of pollutants in sewage has garnered significant attention from researchers, prompting the exploration of various treatment methods, including flocculation precipitation [8], adsorption [9,10,11], and biological approaches [12]. While these methods have shown effectiveness, they are not without limitations. Flocculation precipitation methods suffer from relatively poor treatment efficacy for specific pollutants and the generation of substantial sludge, leading to high treatment costs. Adsorption methods face constraints due to the adsorbent’s saturation capacity and regeneration difficulties, with certain pollutants proving less amenable to adsorption. Biological methods may experience influence from environmental factors such as temperature and pH, resulting in slower treatment rates. In contrast, semiconductor photocatalysis offers a direct route to oxidize and degrade pollutants via generating photoinduced electrons and holes, hence exhibiting a high degradation efficiency [13,14,15]. Moreover, photocatalysts can optimize their performance through surface modification and carrier design, functioning within the visible light range, thus broadening the scope of photocatalytic applications and imparting this technology with enhanced potential and advantages compared to traditional methods.

The origins of photocatalysis can be traced back to 1972 when Fujishima et al. reported that TiO_2_ can decompose water into O_2_ and H_2_ under ultraviolet (UV) irradiation [16]. This discovery has since garnered significant attention from scientists. Semiconductor photocatalysis has subsequently achieved significant breakthroughs in the fields of environment and energy. Photocatalysts are widely used in water treatment due to their high photocatalytic activity, cleanliness, lack of pollution, and stability [17]. However, many photocatalysts, such as TiO_2_ and ZnO, only exhibit activity under UV irradiation due to their wide band gaps [18,19,20,21,22]. This limitation in the light response range severely hampers the utilization of sunlight. To maximize the utilization of solar energy, researchers have developed numerous photocatalysts with visible light activity [23].

**Figure 1:**
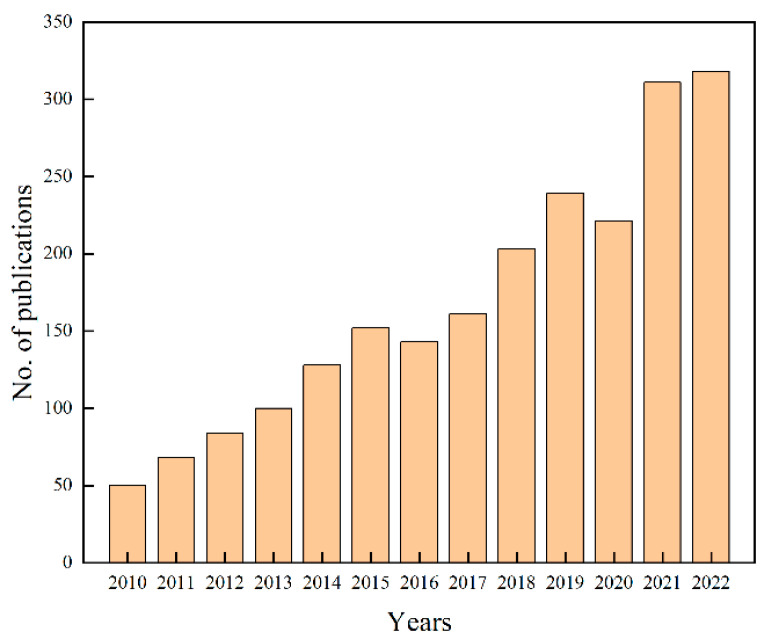
According to data obtained from the Web of Science, the figure displays the number of publications related to the keywords ‘Bi_2_WO_6_’ and ‘photocatalytic’.

Many bismuth-based materials have a narrow band gap due to the hybridization of Bi 6s and O 2p orbitals at the valence band (VB) maximum. This property makes them suitable as visible light responsive photocatalysts [24,25,26]. One such material, Bi_2_WO_6_, which belongs to the Aurivillius phase family, has garnered considerable attention due to its unique layered structure, favorable visible light photocatalytic activities, high thermal and photochemical stabilities, and environmental friendliness [27,28]. It has also been used in water pollution treatment to remove various pollutants [29,30]. To date, Bi_2_WO_6_ has been synthesized using various methods, such as the sol-gel, co-precipitation, molten salt, solvothermal, and hydrothermal processes [31,32,33]. These different synthetic methods have led to the study of numerous Bi_2_WO_6_-based photocatalytic materials with distinct properties in environmental, energy, and biological fields [34,35,36,37,38,39]. However, standalone Bi_2_WO_6_ as a photocatalyst has certain limitations, including rapid recombination of photogenerated electrons and holes, as well as poor photocatalytic activity. To overcome these shortcomings, several modification strategies have been proposed, including element doping [40], metal deposition [41], and heterojunction construction [42]. These modifications enhance the photocatalytic performance of Bi_2_WO_6_ and enable its application in degrading various pollutants such as dyes, antibiotics, and bacteria [43,44,45]. Despite the progress in modifying Bi_2_WO_6_ for photocatalytic applications, a detailed review of its modification strategies, specifically for water treatment purposes, has not been conducted. Importantly, research on Bi_2_WO_6_ in this area is growing rapidly (Figure 1). Reviewing the water pollution treatment applications of Bi_2_WO_6_ based on different modification strategies is necessary to foster further advancements in the field.

This review starts by introducing the structure and property characteristics of Bi_2_WO_6_. We then shift our focus to reviewing different modification strategies that have been used to enhance the photocatalytic performance of Bi_2_WO_6_. These strategies encompass morphology control, metal and non-metal doping, semiconductor heterojunction, and defective engineering. Furthermore, this review explores recent advancements in water pollution treatment and related applications, as well as delving into the photocatalytic mechanism of Bi_2_WO_6_-based photocatalysts. Additionally, we discuss the current challenges and propose potential research directions for future Bi_2_WO_6_-based photocatalysts.

## 2. Structure, Property Characteristics, and Photocatalytic Fundamentals of Bi_2_WO_6_

### 2.1. Structure and Property Characteristics

**Figure 2:**
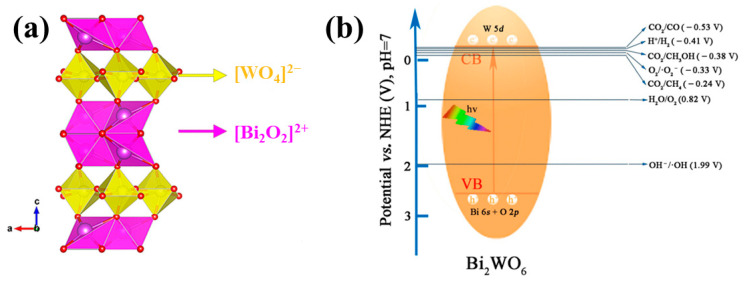
Illustrates important features of Bi_2_WO_6_ [47]. (**a**) It presents the crystal structure of Bi_2_WO_6_ [51]. (**b**) The figure showcases the redox potentials of different species and the energy band positions of Bi_2_WO_6_.

Bi_2_WO_6_ is a relatively straightforward Aurivillius oxide, featuring an orthorhombic structure that consists of corner-shared [WO_4_]^2−^ layers connected to [Bi_2_O_2_]^2+^ layers [46]. Figure 2a displays the structure of Bi_2_WO_6_, where the interleaved layers create an internal electric field that enables the efficient separation of photogenerated electrons and holes [47]. It is well known that the conduction band (CB) of Bi_2_WO_6_ is composed of W 5d orbitals, while the valence band (VB) is predominantly made up of the hybrid orbitals of Bi 6s and O 2p [48]. With a band gap of approximately 2.7 eV, Bi_2_WO_6_ is a visible light responsive photocatalyst [49,50]. Figure 2b provides insight into some thermodynamic data associated with the photocatalytic reaction process. Remarkably, Bi_2_WO_6_ exhibits a positive VB, indicating its significant capability to degrade a broad range of pollutants [51].

### 2.2. Fundamentals of Photocatalysis

**Figure 3:**
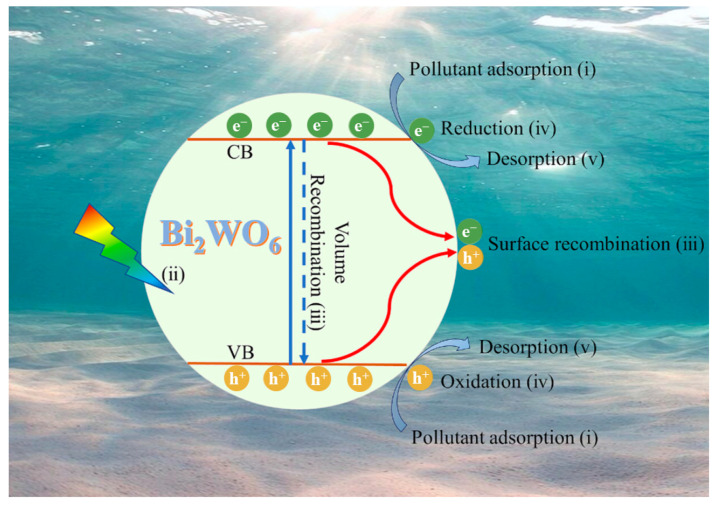
Schematic of photocatalytic pollutants degradation on the Bi_2_WO_6_ catalysts [52].

Figure 3 illustrates the photocatalytic reaction mechanisms of Bi_2_WO_6_ catalysts [52].

The mechanisms can be summarized as follows: Pollutants are adsorbed on the catalyst surface. When the catalyst absorbs photon energy higher than the band gap energy, photoinduced electrons move from the valence band (VB) to the conduction band (CB), creating holes in the VB. The electrons (e^−^) and holes (h^+^) generated migrate to the catalyst’s surface. However, only a small portion (less than 10%) of the generated carriers is available for the photocatalytic reaction, with approximately 90% of them recombining. The catalyst-captured energy activates groups such as superoxide radicals (·O_2_^−^) and hydroxyl radicals (·OH), utilizing the generated electrons and holes from the photocatalyst. These radicals are responsible for the degradation of pollutants. Moreover, the electrons and holes themselves can directly degrade pollutants. The degraded products are released from the catalyst’s surface, allowing the photocatalytic reaction to continue. By facilitating the degradation of pollutants and enabling the continuation of the photocatalytic reaction, the Bi_2_WO_6_ catalysts play a crucial role in the process.

The photocatalytic efficiency of materials is influenced by several critical factors. These factors include the light absorption ability of the photocatalysts, which is determined by the bandgap of the semiconductor [53], and the separation and transfer efficiencies of the photogenerated electron-hole pairs. High carrier recombination rates result in low photocatalytic efficiency [54]. Furthermore, the external photocatalytic reaction conditions significantly impact the photocatalytic efficiency of the photocatalysts. Factors such as the pH value of the solution, photocatalyst dosage, reaction time, and initial concentrations of pollutants all play vital roles. In a photocatalysis experiment, the pH of the solution can influence the chemical forms of pollutants present in water. Many pollutants exist in different ionic states under varying pH conditions, impacting their reactivity, solubility, and interaction with photocatalysts. Modifying the solution’s pH can alter the charge state of pollutants, thereby affecting their solubility and adsorption properties in water, consequently influencing the progression of the photocatalytic reaction. Moreover, pH variations can also affect the dispersion stability of particles in the solution, consequently impacting the particles’ zeta potential. Unstable dispersion may result in particle aggregation, reducing the effective surface area of the photocatalyst and potentially compromising the efficiency of the photocatalytic reaction. Therefore, achieving efficient photocatalytic reactions requires the use of appropriate photocatalysts and the establishment of suitable reaction conditions.

However, the Bi_2_WO_6_ catalyst faces several challenges as a photocatalyst. Firstly, it can only absorb visible light with wavelengths below 450 nm, which is due to its band gap limitation. This restriction hinders its potential for efficiently utilizing the entire visible light spectrum. Secondly, the recombination rate of the photogenerated electron-hole pairs in the Bi_2_WO_6_ catalyst remains high, resulting in a decrease in overall photocatalytic efficiency. Additionally, the Bi_2_WO_6_ catalyst lacks a sufficient number of surface active sites, which limits its capacity for carrying out photocatalytic reactions. Therefore, it is crucial to optimize these properties of the Bi_2_WO_6_ catalyst in order to enhance its photocatalytic activity. This can be achieved through various modification methods, including morphology control, metal and non-metal doping, and the formation of semiconductor heterojunctions [55,56].

## 3. Morphology Control

**Figure 4:**
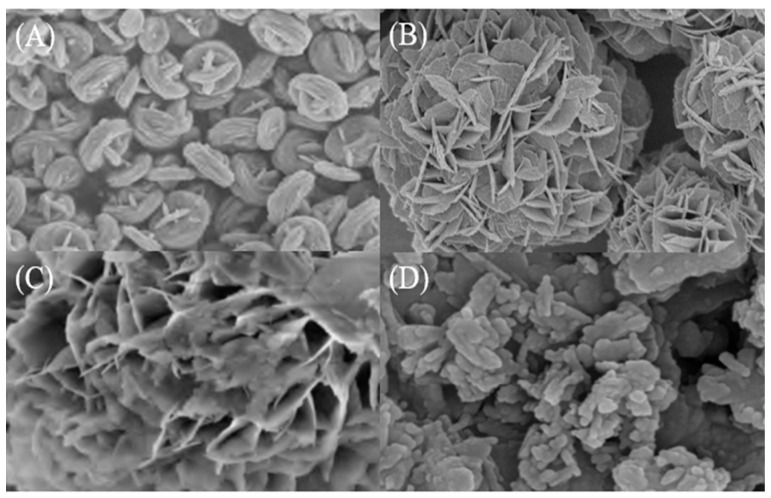
SEM images of (**A**) knob-like [57], (**B**) rose-like [58], (**C**) nanosheets [59], and (**D**) powder [60] Bi_2_WO_6_ catalyst.

**Figure 5:**
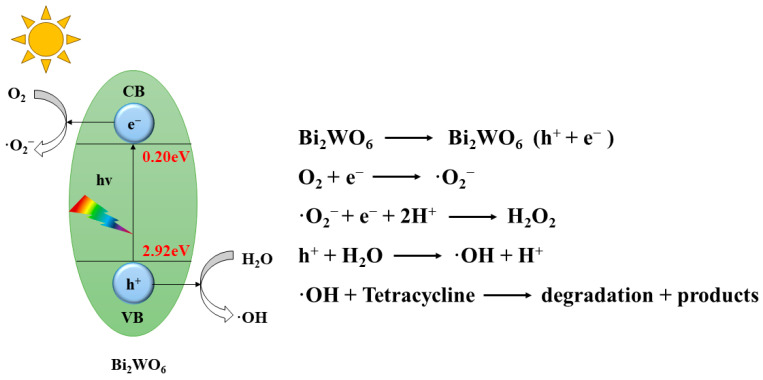
Mechanism of photocatalytic degradation of tetracycline by Bi_2_WO_6_ catalyst [61].

The performance of photocatalytic materials depends not only on their chemical composition, but also on their size and shape. In the case of the Bi_2_WO_6_ catalyst, its morphology is influenced by the method of synthesis used, such as precipitation, hydrothermal, or solvothermal methods, which are commonly employed in its preparation. These methods often result in irregularly shaped Bi_2_WO_6_ particles. It is advantageous to have a high specific surface area with numerous active sites, as this enhances the catalyst’s ability to adsorb reactants. The crystal structure and morphology of Bi_2_WO_6_ can be controlled by adjusting synthesis conditions and incorporating surfactants. These factors, in turn, affect its specific surface area, light absorption performance, and efficiency in separating photogenerated carriers. The morphology of the Bi_2_WO_6_ catalyst generally falls into four categories: three-dimensional (flower-like), two-dimensional (nanoplates), one-dimensional (nanofiber), and zero-dimensional (nanoparticles). These categories are summarized in Table 1. For example, in Figure 4, Guo et al. [57] and Zheng et al. [58] achieved knob-like and rose-like morphologies of Bi_2_WO_6_ photocatalytic materials using the hydrothermal method. Wang et al. [59] obtained a Bi_2_WO_6_ photocatalytic material consisting of nanosheets with a large specific surface area through hydrothermal synthesis. Zhou et al. [60] prepared Bi_2_WO_6_ powder using the solid phase method, and investigated the impact of different reaction temperatures on its activity. When the preparation temperatures were 300 °C, 350 °C, and 400 °C, the adsorption rates of the samples for Rhodamine B (RhB) solution were 31%, 22%, and 10%, respectively. The gradual decrease in adsorption can be attributed to the gradual increase in particle size and reduction in specific surface area of the powder. As the semiconductor photocatalytic reaction primarily occurs on the surface of the photocatalyst powder, a larger specific surface area is advantageous for increased RhB adsorption, thereby promoting photocatalytic degradation. At a preparation temperature of 400 °C, the resulting powder exhibited significantly increased grain size, leading to reduced photocatalytic activity, with a RhB photodegradation rate of only 47%. On the other hand, at 300 °C, despite the highest adsorption rate of RhB by the obtained powder, its photocatalytic activity was lower than that of the powder obtained at 350 °C. This may be due to the better crystallinity of the powder obtained at 350 °C. Increased semiconductor crystallinity can reduce crystal defects, which are often the recombination centers for photogenerated electrons and holes. Overall, the tungsten bismuth oxide powder obtained at 350 °C demonstrated the strongest visible light photocatalytic activity, achieving a RhB photodegradation rate of 98% after visible light irradiation for 120 min. Mei et al. [61] synthesized nanoplate Bi_2_WO_6_ catalysts via the hydrothermal method. With increased hydrothermal synthesis time, the thickness of the nanoplates decreased and their crystallinity became more stable. When the pH is low (pH < 8), BWO-8/BWO-T-10 nanoplates exhibited the highest photocatalytic activity. After two hours of light exposure, the degradation efficiency of tetracycline reached 85%, with a reaction rate of 0.0135 min^−1^. The material remained highly stable even after three cycles of repeated use. The catalytic mechanism analysis is depicted in Figure 5.

## 4. Metal Doping

Metal doping refers to the process of introducing metal elements into a photocatalyst to enhance its properties. In the synthesis of the Bi_2_WO_6_ catalyst, metals can be added to the reaction system. Metal doping can change the band structure of a semiconductor by introducing additional energy levels or adjusting the lattice structure. This alteration can impact the optoelectronic properties of the semiconductor, such as changes in absorption spectra and the lifetime of photogenerated carriers, thereby influencing its photocatalytic activity [68]. It can also improve the capacity of the photocatalyst to degrade organic pollutants. Additionally, certain metal ions can induce the formation of surface defects, such as oxygen vacancies, through charge compensation mechanisms resulting from the difference in valence between the dopant and the parent cation. It is important to carry out metal doping in a reasonable manner according to the specific requirements of each application. For example, Zhu et al. [69] prepared a Sn-doped Bi_2_WO_6_ catalyst, which showed a larger specific surface area and more active sites. The degradation rate of methylene blue increased by 11.4%, and the degradation rate of methylene blue reached 92% when the doping rate of Sn was 2%. Bunluesak et al. [70] constructed an Ag-doped Bi_2_WO_6_ catalyst and studied its degradation efficiency on RhB. The incorporation of silver ions can effectively enhance the interfacial charge diffusion and photocatalytic activity of nanoplates. The results showed that the degradation efficiency of pure Bi_2_WO_6_ and 10%Ag/Bi_2_WO_6_ on RhB were 47.79% and 94.21% under the visible light irradiation, respectively. Gao et al. [71] prepared a Cu-doped Bi_2_WO_6_ photocatalyst using a hydrothermal method. The Cu-Bi_2_WO_6_ displayed a three-dimensional flower spherical structure with a large specific surface area (85 m^2^/g), and exhibiting high photocatalytic activity. When the pH was 6 and the Cu load was 0.5 wt.%, the degradation efficiency of phenol by the composite was as high as 92%, 23% higher than Bi_2_WO_6_. Zhu et al. [72] also constructed Mg^2+^, Fe^3+^, Zn^2+^, and Cu^2+^ doped Bi_2_WO_6_ catalysts, which exhibited excellent photodegradation performances for antibiotics. Among these catalysts, the Mg^2+^ doped Bi_2_WO_6_ catalyst showed the highest degradation rates of 89.44 and 99.11% for norfloxacin (NOR) and ciprofloxacin (CIP), respectively. And the specific surface area of Mg/Bi_2_WO_6_ was 1.6 times higher than Bi_2_WO_6_. Other metals can also be added to the Bi_2_WO_6_ photocatalysts as shown in Table 2. Metal doping can significantly affect the photocatalytic activity of Bi_2_WO_6_, altering its performance in photodegradation processes.

## 5. Non-Metal Doping

The distribution of metal ions on the surface of metal-doped photocatalysts can be uneven, leading to aggregation or dispersion, which affects the material’s overall performance. Non-metals generally have high ionization energy and electronegativity. When incorporated as dopants, non-metal elements primarily impact the electronic structure and redox properties of the Bi_2_WO_6_ catalyst. Non-metals can alter the band structure in the Bi_2_WO_6_ catalyst by changing the electron affinities and ionization energies [83]. Additionally, non-metal doping allows for high control precision, accurate concentration, and distribution, making it suitable for modifying Bi_2_WO_6_ photocatalysts and enhancing photocatalytic efficiency [84]. For instance, Li et al. [85] prepared flower-like bismuth tungstate using a hydrothermal method with carbon as the carrier. The modified bismuth tungstate catalyst exhibited an excellent photocatalytic performance, with degradation rates of RhB and tetracycline reaching 98% and 87%, respectively. This is attributed to the fact that Bi_2_WO_6_/C (2.35 eV) has a narrower band gap than Bi_2_WO_6_ (2.64 eV), and the large-area flower-like Bi_2_WO_6_. Zhang et al. [86] synthesized I-doped Bi_2_WO_6_ photocatalysts through a hydrothermal method, and the amount of iodine doping influenced the photocatalytic activity of the catalysts. The study revealed that 1.0 wt.% iodine-doped Bi_2_WO_6_ showed the best removal effect on Hg under visible light irradiation, achieving 97.5% efficiency. The mercury-removal efficiency of Bi_2_WO_6_ was 9.1%. This is attributed to the reduction in the potential energy of the conduction band and the decrease in electron-hole pair recombination rate. To enhance the photocatalytic activity of the catalysts, Zheng et al. [87] precipitated silicon carbide, which has a high electron transfer rate, onto bismuth tungstate using a hydrothermal method. This yielded a RhB degradation rate 3.7 times higher than pure bismuth tungstate. Zhu et al. [88] employed a simple hydrothermal method to modify bismuth tungstate with S. The addition of S can effectively change the morphology of bismuth tungstate, resulting in a rose-shaped S/Bi_2_WO_6_ composite material, with a Rhodamine B degradation rate of 96.2%. Non-metal doping can influence the morphology, band gap width, and electron-hole recombination rate of bismuth tungstate, thereby enhancing its photocatalytic performance (see Table 3).

## 6. Semiconductor Heterojunction

**Figure 6:**
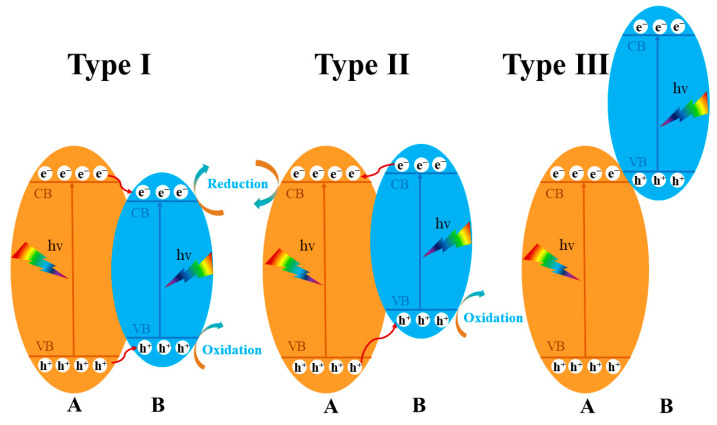
Schematic illustration of the three different types of semiconductor heterojunction photocatalysts.

A heterojunction is constructed by combining different materials and adjusting their band gap, electrical conductivity, and optical properties. For example, using bismuth tungstate and other semiconductors can effectively change the electronic structure and separate photogenerated charges. Semiconductor heterojunction photocatalysts consist of two or more semiconductors with different energy band structures connected by tightly bound interfaces. Traditionally, there are three types of semiconductor heterojunctions: type I, type II, and type III (see Figure 6). Type I heterojunctions have a nested band structure where the conduction band (CB) position of semiconductor A is higher than that of semiconductor B, but its valence band (VB) level is lower. Unfortunately, this structure causes photogenerated electrons and holes to migrate and aggregate to the semiconductor with the smaller band gap, reducing the redox potential of the photocatalyst and hindering charge separation. Type II heterojunctions, on the other hand, have an ecotone structure in which the CB and one of the semiconductors have higher VB levels than the other. This difference means that the electrons and holes move in opposite directions, allowing for spatial separation and efficient charge separation. Type III heterojunctions have disjointed band structures that prevent carriers from moving between semiconductors, making efficient charge separation impossible. Most research on traditional heterojunctions has focused on type II junctions [93,94].

**Figure 7:**
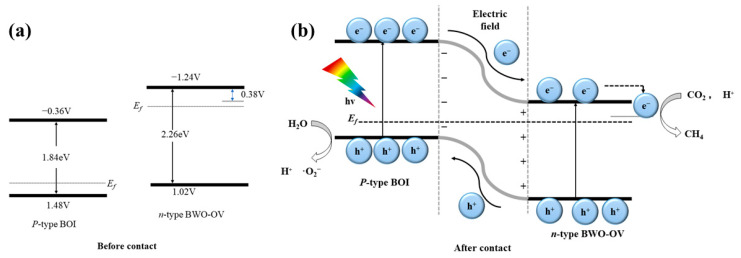
(**a**) Band positions of BOI and Bi_2_WO_6_-OV. (**b**) The process of charge transfer and separation of p-n heterojunction.

The semiconductor p-n junction is a special heterostructure composed of p-type and n-type semiconductors that allows for efficient charge separation [95,96]. Kong et al. [97] synthesized a novel 2D Bi_2_WO_6_/BiOI catalyst with a p-n junction and surface oxygen vacancies. The mechanism of this p-n junction is depicted in Figure 7. The Fermi level (Ef) of Bi_2_WO_6_ (n-type semiconductor) is higher than that of BiOI (p-type semiconductor). Consequently, when they come into contact, electrons diffuse from Bi_2_WO_6_ to BiOI, while holes diffuse from BiOI to Bi_2_WO_6_. This results in a lowering of the energy band of Bi_2_WO_6_ and an upward shift of BiOI’s Ef, until the system reaches an equilibrium, resulting in the formation of an interfacial electric field from Bi_2_WO_6_ to BiOI. Under visible light irradiation, photogenic electrons and holes are generated in Bi_2_WO_6_ and BiOI. Thanks to the internal electric field, holes in Bi_2_WO_6_ quickly transfer to BiOI, while the electron transfer path is reversed. Ultimately, the p-n heterostructure enables ultra-fast directional migration and spatial separation of photogenerated carriers. Furthermore, there exists a multitude of semiconductor heterojunction photocatalysts such as p-n junction Bi-OI/Bi_2_WO_6_ [98], Co_3_O_4_/Bi_2_WO_6_ [99], CoO/Bi_2_WO_6_ [100], Bi_2_WO_6_/CuS [101], and CuA-lO_2_/Bi_2_WO_6_ [102] that have been utilized for the degradation of environmental pollutants. Mao et al. [101] successfully prepared a Bi_2_WO_6_/CuS photocatalyst through the hot solvent method, which exhibited outstanding degradation rates of 99.9%, 74.7%, and 75.7% for single Rhodamine B, tetracycline, and Cr (VI) solutions, respectively. In mixed solutions, the degradation rates of Rhodamine B, tetracycline, and Cr (VI) were 97.7%, 87.6%, and 95.1%, respectively. Moreover, when multiple wastewaters co-exist, p-n heterojunctions in Bi_2_WO_6_/CuS shorten the electron transport path, effectively separating and transferring photoelectrons and holes, thereby improving the removal efficiency of both pollutants. In another study by Xie et al. [103], a layered MoSe_2_/Bi_2_WO_6_ composite photocatalyst was prepared using the ultrasonic method. MoSe_2_/Bi_2_WO_6_ showed high catalytic activity under visible light irradiation for 3 h, achieving a degradation rate of p-toluene of nearly 80%. This was attributed to the formation of a p-n heterojunction between materials, which can inhibit the recombination of electron-hole pairs, increase the content of superoxide and hydroxyl free radicals on the surface, and enhance the photocatalytic process.

**Figure 8:**
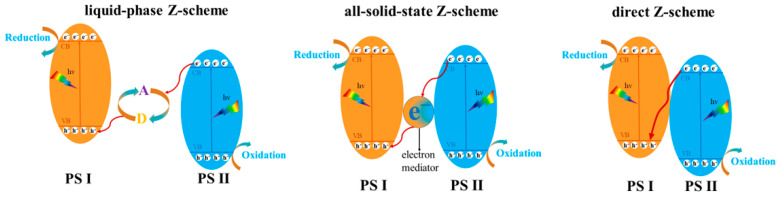
Schematic illustration of the liquid-phase Z-scheme system, the all-solid-state Z-scheme system, and the direct Z-scheme system.

Traditional type II semiconductor heterojunctions can improve the separation efficiency of photogenerated carriers. However, the directional migration of photogenerated electrons to the corrected CB and holes to more negative VB can reduce the redox potential of the original photogenerated electrons and holes. Additionally, repulsive forces arise between identical charges, which effectively inhibit the electrons and holes to migrate and accumulate. In order to address these challenges, Z-schemes like plant photosynthesis have gained significant attention. As shown in Figure 8, the development history of Z-scheme photocatalysts can be divided into three generations: the liquid-phase Z-scheme, the all-solid-state Z-scheme, and the direct Z-scheme junctions [104]. Furthermore, heterojunctions such as S-type heterojunctions can also be formed between Bi_2_WO_6_ and other semiconductor substances. Table 4 lists some of the typical Z-scheme and S-scheme junctions.

## 7. Defect Engineering

**Figure 9:**
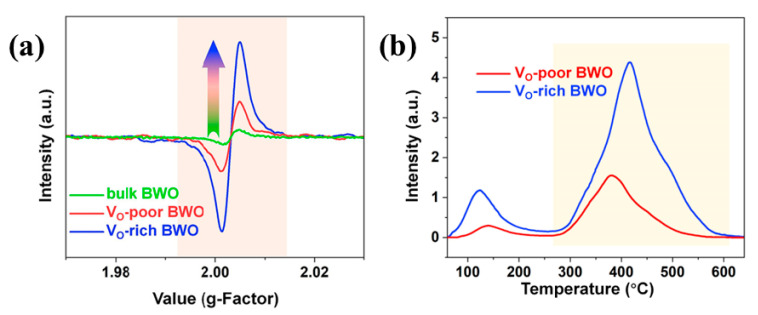
Electron paramagnetic resonance spectra of bulk Bi_2_WO_6_, VO-poor Bi_2_WO_6,_ and VO-rich Bi_2_WO_6_ (**a**); O_2_ inneiring teracts with the (**b**) VO-rich Bi_2_WO_6_ and VO-poor Bi_2_WO_6_.

Defect engineering is a promising technique to improve the photocatalytic performance of Bi_2_WO_6_ photocatalysts by modifying their crystal defects. Crystal defects are crucial in altering the properties of the photocatalytic materials. These defects refer to the disruption of the periodic arrangement of atoms or molecules in the crystal material and are present in all photocatalysts, thus affecting their photocatalytic behavior. Crystal defects can be classified based on their size into zero-dimensional point defects, such as vacancies, one-dimensional line defects, such as edge dislocations, two-dimensional planar defects, such as grain boundaries, and three-dimensional bulk defects, such as disordered regions [117]. Crystal defects can also be categorized into point defects, line defects, surface defects, and block defects based on their respective positions. Recently, the focus of defect engineering in Bi_2_WO_6_ photocatalysts has been on vacancy defects. Gao et al. [118] prepared Bi_2_WO_6_ ultrathin nanosheets with an abundance of oxygen vacancies. The presence of oxygen vacancies in the Bi_2_WO_6_ structure was confirmed through electron paramagnetic resonance spectroscopy (Figure 9a). The presence of vacancy defects in Bi_2_WO_6_ photocatalysts has a significant impact on their photocatalytic activity and performance. Density functional theory indicates that Bi_2_WO_6_ with an abundance of oxygen vacancies has a higher adsorption energy, resulting in a stronger capacity to attract and adsorb oxygen molecules (Figure 9b). This suggests that oxygen vacancies enhance the material’s ability to capture oxygen. Additionally, Hang et al. [119] developed Bi_2_WO_6_ photocatalysts with both tungsten (W) and oxygen (O) vacancies. The introduction of these vacancies causes a decrease in the valence band (VB) potential, promoting the generation of highly oxidizing holes. Furthermore, these vacancies trap charges and facilitate the separation of electron-hole pairs, ultimately enhancing the photocatalytic performance. Overall, this study highlights the role of vacancies in optimizing the photocatalytic performance of Bi_2_WO_6_.

## 8. Bi_2_WO_6_ Photoelectrochemistry

Photoelectrochemistry (PEC) mainly consists of two processes: photoelectroconversion and electrochemistry. Photoelectroconversion refers to the electron transition in light-active materials upon photon absorption, leading to the generation of photoinduced charges, followed by charge separation and transfer, resulting in the formation of photovoltage and the conversion of light energy into electrical energy [120].The electrochemical process involves the partial separation of charges transferring to the electrode/solution interface, where oxidation-reduction reactions occur, generating electrical signals and converting chemical energy into electrical energy. In the photoelectrochemical process, light-active materials serve as the basis of the reaction. Typically, these materials transfer electrons from the conduction band to the electrode, while the holes in the valence band undergo oxidation reactions with electron donors in the solution [121]. Wang et al. [122] reported a hybrid of Bi_2_WO_6_ wrapped with reduced graphene oxide (Bi_2_WO_6_@rGO) as a photoelectrode for enhanced photocatalytic degradation of organic pollutants. The Bi_2_WO_6_@rGO hybrid exhibited a 43.0% and 65.6% increase in the photocatalytic degradation efficiency of Rhodamine B compared to the photocatalytic and electrocatalytic processes, respectively. The enhancement in the photoelectrocatalytic degradation of RhB in the Bi_2_WO_6_@rGO hybrid is attributed to a negative shift of 0.26 V in the flat band potential and the spatial separation of photogenerated electrons and holes by external potentials. Pedanekar et al. [123] successfully deposited Bi_2_WO_6_ thin films using a simple spray pyrolysis technique. The film deposited with a 70 mL spraying solution quantity exhibited a higher photocurrent density (460 mA/cm^2^), and the same film, with a large area (10 × 10 cm^2^), was used for the photocatalytic and photoelectrocatalytic degradation of Rhodamine B dye under solar radiation. The photoelectrocatalytic removal of RhB exhibited a higher degradation efficiency (94%) compared to the photocatalytic process (23%).

## 9. Conclusions and Outlook

In this review, we provide a comprehensive summary of the recent progress made in the field of water treatment using Bi_2_WO_6_-based photocatalysts. We begin by briefly outlining the basic properties and photocatalytic fundamentals of Bi_2_WO_6_ photocatalysts. Next, we delve into a detailed discussion of various strategies employed to regulate the performance of Bi_2_WO_6_ photocatalysts, including morphology control, metal and non-metal doping, semiconductor heterojunction, and defective engineering. Through the implementation of these strategies, we are able to greatly enhance the light absorption capabilities and separation efficiency of photogenerated electrons and holes. Additionally, we can regulate the surface properties of Bi_2_WO_6_, effectively bolstering the overall photocatalytic performance of these materials. The modified Bi_2_WO_6_ photocatalysts showcased remarkable degradation efficiencies for organic dyes, antibiotics, and bacteria in aqueous environments. In conclusion, achieving efficient Bi_2_WO_6_-based photocatalysts requires several key factors to be carefully considered. Firstly, controlling the hydrothermal synthesis conditions, such as adjusting the pH value and reaction temperature of the precursor solution, can significantly affect the morphology and performance of the catalyst. Secondly, choosing a suitable dopant can alter the band structure, extend the photo response range of the catalyst, and improve its overall performance. Thirdly, leveraging the local surface plasmon resonance effect by loading appropriate metals onto the catalyst surface promotes effective charge separation, thus enhancing catalytic activity. Fourthly, producing Bi_2_WO_6_ heterostructures with other photocatalysts with matching energy bands can create synergistic effects and widen the range of degradable pollutants. Finally, creating surface defects, such as the introduction of oxygen vacancies, play a crucial role in trapping electrons and facilitating effective carrier separation, ultimately improving catalytic performance.

However, despite these improvements, obstacles and challenges remain in the research of Bi_2_WO_6_-based photocatalysts. These limitations must be overcome to fully utilize the benefits of Bi_2_WO_6_ in the field of wastewater purification. Firstly, controlling the morphology of Bi_2_WO_6_ during synthesis is typically achieved through adjusting solution pH and utilizing surfactants. However, regulating the catalytic active site remains imprecise. Furthermore, further exploration of crystal surface control methods can improve the photocatalytic activity of Bi_2_WO_6_. Secondly, the current research mostly concentrates on pollutant degradation in water environments. Nonetheless, water environments contain various types of contaminants including, but not limited to, pesticides, radioactive substances, toxic algae, and harmful organic and inorganic materials. Interactions between different pollutants can heighten the toxicity of the water body. Therefore, assessing the photocatalytic capacity of different photocatalysts in water with co-existing pollutants is necessary. Thirdly, optimizing photocatalysts to facilitate the separation of photogenerated carriers has been a primary focus. The Z-scheme heterojunction effectively separates carriers, enhances the reduction capacity of Bi_2_WO_6_, and preserves the negative electrons and positive holes. Therefore, Z-scheme heterojunctions can be achieved by combining Bi_2_WO_6_ with narrow-band gap semiconductors with a more negative CB. However, there is limited research in these areas, and the inclusion of external fields is expected to become a prominent research focus in the future. Although there have been significant developments in the preparation and degradation applications of Bi_2_WO_6_ in recent years, most studies have been conducted on a laboratory scale due to the unstable photocatalytic properties of Bi_2_WO_6_ in large-scale applications. This issue needs to be prioritized, as large-scale industrial applications of photocatalysts are crucial for environmental contaminant removal. Considering the practical applications, the toxicity of Bi_2_WO_6_-based catalysts is an important consideration. However, overall, Bi_2_WO_6_-based photocatalysts are universally recognized as green. Yet, it is important to note that Bi_2_WO_6_-based photocatalysts can generate reactive oxygen species and free radicals during their photocatalytic processes. These reactive species have the potential to cause cellular toxicity. Therefore, further research is necessary to fully understand the potential toxicity of Bi_2_WO_6_ nanomaterials, especially their effects on human health. It is critical to conduct comprehensive biosafety experiments and studies to assess the potential risks associated with the use of Bi_2_WO_6_-based photocatalysts. This will help ensure the safe and responsible use of Bi_2_WO_6_ nanomaterials in a variety of applications.

## Figures and Tables

**Table 1:** Morphology regulation of bismuth tungstate photocatalyst.

Pollutants	Morphology	Precursor	Method	Efficiency	Reference
methylbenzene	Nest-like	Bi(NO_3_)_3_·5H_2_O Na_2_WO_4_	hydrothermal	100%	[62]
tetracycline	Flower-like	Na_2_WO_4_·2H_2_O Bi(NO_3_)_3_·5H_2_O	hydrothermal	88%	[63]
tetracycline	Nanosheet	(NH_4_)_10_H_2_(W_2_O_7_)_6_ Bi(NO_3_)_3_·5H_2_O	hydrothermal	70%	[64]
tetracycline hydrochloride	Hollow microspheres	Na_2_WO_4_·2H_2_O Bi(NO_3_)_3_·5H_2_O	precipitation	95%	[65]
RhB	Tube-like	Bi_2_O_3_ Na_2_WO_4_·2H_2_O	solvothermal	99%	[66]
RhB	Nanofiber	Na_2_WO_4_·2H_2_O Bi(NO_3_)_3_·5H_2_O	electrospinning	71%	[67]

**Table 2:** Metal doping of bismuth tungstate photocatalysts.

Photocatalyst	Method	Modified Band Gap Value	Pollutants	Efficiency	Reference
Pd/Bi_2_WO_6_	hydrothermal	/	RhB	99.33%	[73]
MIL-53(Fe)/Bi_2_WO_6_	hydrothermal	2.61 eV	phenol	98%	[74]
Mn-doped Bi_2_WO_6_/GO/MoS_2_	hydrothermal ultrasonic	2.2 eV	methylene blue	99%	[75]
Ni/Bi_2_WO_6_	hydrothermal	2.74 eV	RhB	93%	[76]
Ni/Ti-Bi_2_WO_6_	hydrothermal	2.89 eV	tetracycline	92.9%	[77]
Pt/Bi_2_WO_6_	precipitation	3.08 eV	RhB	98.09%	[78]
Cd-Bi_2_WO_6_	hydrothermal	2.58 eV	RhB	100%	[79]
Au-Bi_2_WO_6_	hydrothermal	2.96 eV	RhB	96.25%	[80]
Ce-Bi_2_WO_6_	hydrothermal	2.23~2.26 eV	salicylic acid	91.6%	[81]
In-Bi_2_WO_6_	sol-gel	2.74 eV	RhB	90%	[82]

**Table 3:** Non-metal doping of bismuth tungstate.

Doping Element	Method	Modified Band Gap Value	Pollutants	Efficiency	Reference
I	solvothermal microwave	2.29 eV	RhB	99%	[89]
Br	hydrothermal	0.916 eV	RhB	95%	[90]
rGO	hydrothermal	/	norfloxacin	87.49%	[91]
N	hydrothermal microwave	/	RhB	81%	[92]

**Table 4:** Heterostructure of bismuth tungstate photocatalyst.

Heterostructure	Catalyst	Method	Pollutants	Efficiency	Reference
S-scheme	Bi_2_WO_6_/CoIn_2_S_4_	hydrothermal	tetracycline	90%	[105]
	Bi_2_WO_6_/Bi_2_O_3_	trituration	nitrobenzene	95.7%	[106]
	Bi_2_WO_6_/Fe_2_O_3_/WO_3_	ultrasonic immersing	bisphenol A	99%	[107]
	CdS@Bi_2_WO_6_	solvothermal	RhB	96.1%	[108]
Z-scheme	Bi_2_WO_6_/BiOBr/rGO	hydrothermal	norfloxacin	95.12%	[109]
	SnS/Zn_2_SnO_4_	hydrothermal	methylene blue	94.5%	[110]
	Au@TiO_2_/Bi_2_WO_6_	sol-gel	tetracycline	95%	[111]
	Bi_2_WO_6_/C_3_N_4_/TiO_2_	sol-gel	phenol	84.7%	[112]
	g-C_3_N_4_/Gd/Bi_2_WO_6_	hydrothermal	methylene blue	92%	[113]
	BiO_2−x_/Bi_2_WO_6_	solvothermal	phenol	90%	[114]
	polypyrrole/Bi_2_WO_6_	precipitation	Cr (VI)	99.7%	[115]
	AgIn_5_S_8_/Bi_2_WO_6_	solvothermal	Cr (VI)	92%	[116]

## Data Availability

The data presented in this study are available upon request from the corresponding author. The data are not publicly available due to ethical considerations.
